# A275 MECHANOSENSITIVE KCA2 + 1.1 CHANNEL MEDIATES INTRALUMINAL DISTENSION-INDUCED CALCIUM INFLUX IN ENTERIC NEURONS

**DOI:** 10.1093/jcag/gwac036.275

**Published:** 2023-03-07

**Authors:** P Wongkrasant, J -B CAVIN, O Balemba, W MacNaughton, K Sharkey

**Affiliations:** 1 Physiology & Pharmacology, University of Calgary, Calgary, Canada; 2 Biological Sciences, University of Idaho, Idaho, United States

## Abstract

**Background:**

The enteric nervous system (ENS) is the extensive neural network within the wall of the gastrointestinal (GI) tract that is essential for the control of gut function. However, the mechanisms whereby the ENS responds to the mechanical forces associated with distension are not well understood, and this has implications for gut function in health and disease.

**Purpose:**

This study aims to determine the mechanisms underlying the responsiveness of enteric neural circuits in the myenteric plexus to intraluminal distension.

**Method:**

Intact segments of the colon from mice expressing a genetically encoded fluorescent calcium reporter specifically in enteric neurons (Wnt-1-GCaMP6 mice) or cholinergic neurons (Chat-GCaMP6 mice) were bathed and luminally perfused with Krebs solution in a custom-designed chamber (35-37°C, 95% O_2 _,5% CO_2_, pH 7.4). Intracellular Ca^2+ ^fluorescence in enteric neurons was visualized by live-cell confocal microscopy and was analyzed using Imaris™ software. Intraluminal distension was accomplished either by increasing luminal perfusion pressure or using colonic preparations containing natural fecal pellets. The degree of intraluminal distension produced by perfusion was defined by the relative distance between identified neurons in preparation and is expressed as a percentage increase in the distance between them. Distension was studied over a range from 0-75%. The degree of distension produced by a fecal pellet in the distal colon is approximately 75%.

**Result(s):**

Intraluminal distension significantly increased intracellular Ca^2+ ^fluorescence in most (>85%) of neurons of the myenteric plexus. When the colon was distended, the response to depolarization by KCl, a general cell depolarization activator, and DMPP, a nicotinic receptor agonist in Wnt1-cre GCaMP6 mice and Chat GCaMP6 mice, respectively was substantially inhibited. To identify how distention induced an increase in intracellular Ca^2+ ^fluorescence and inhibited depolarization, pharmacological inhibitors of mechanosensitive ion channels were used. The nonspecific mechanosensitive channel inhibitor, gadolinium (50 mM) and the Kca_1.1 _channel inhibitor, paxilline (10 mM), both significantly reduced the luminal distension-induced increase in intracellular Ca^2+^ fluorescence. The effect of luminal distension-inhibited cell depolarization by KCl recovered in the presence of paxilline. This was not observed in the Ca^2+ ^responses evoked by DMPP, suggesting that other mechanisms may play a role in the inhibition of neuronal responses in cholinergic enteric neurons.

**Conclusion(s):**

The majority of neurons of the colonic myenteric plexus are mechanosensitive and show an increase in intracellular Ca^2+ ^when the gut is distended. Myenteric neurons do not respond to nicotinic receptor activation when the gut is distended through a mechanism that is dependent on the activity of Kca_1.1_ channel.

**Supported by CIHR**

**Disclosure of Interest:**

None Declared

